# Supplementary material for: A Multi-Trait Association Analysis of Brain Disorders and Platelet Traits Identifies Novel Susceptibility Loci for Major Depression, Alzheimer’s and Parkinson’s Disease
Source: Cells. 2023 Jan 6;12(2):245. doi: 10.3390/cells12020245 (PMC9856280; doi:10.3390/cells12020245)
Supplement: Supplementary file 1 [file cells-12-00245-s001.zip › cells-2086795-supplementary.pdf]

a)

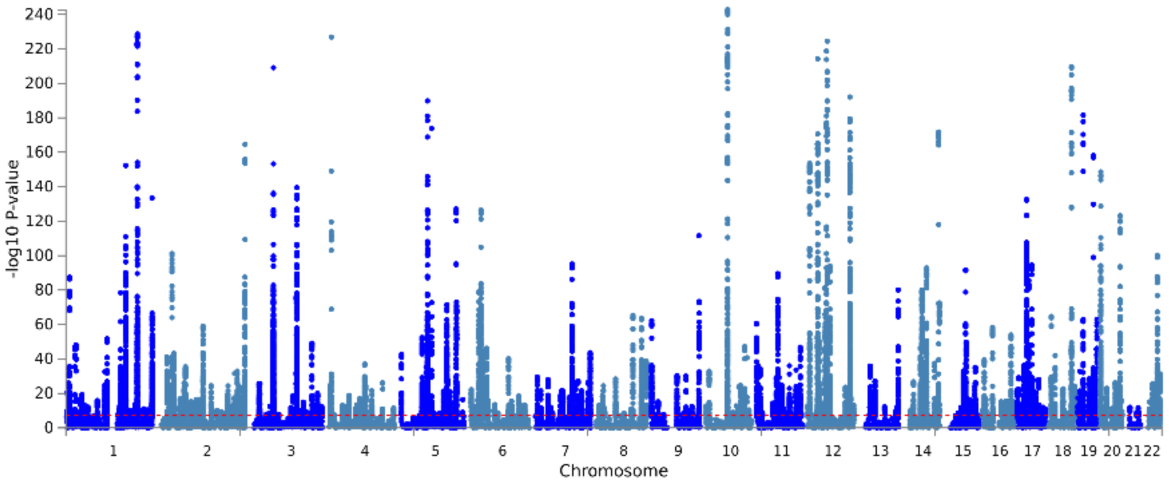
-log10 P-value
240
220
200
180
160
140
120
100
80
60
40
20
0
1 2 3 4 5 6 7 8 9 10 11 12 13 14 15 16 17 18 19 20 21 22
Chromosome

b)

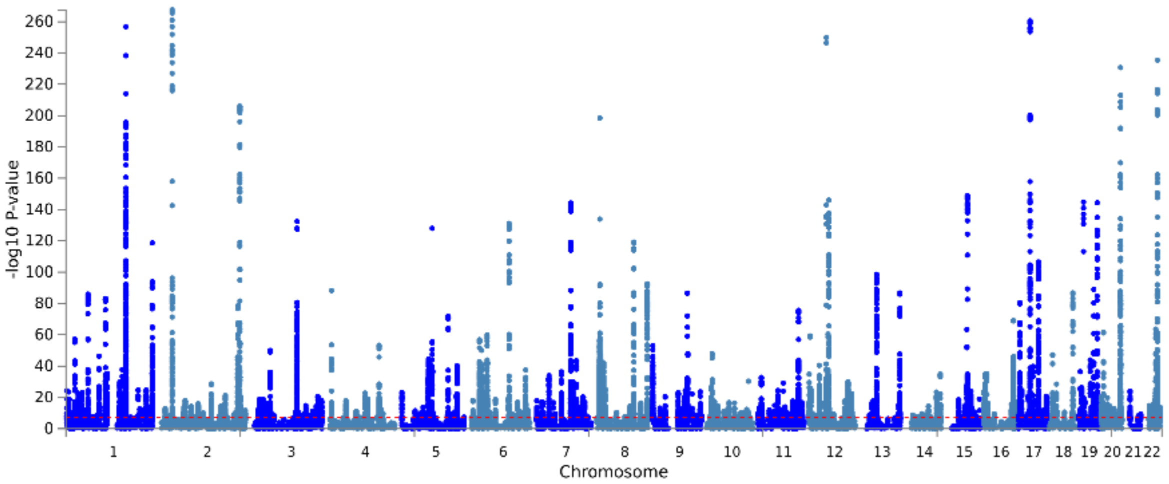
-log10 P-value
260
240
220
200
180
160
140
120
100
80
60
40
20
0
1 2 3 4 5 6 7 8 9 10 11 12 13 14 15 16 17 18 19 20 21 22
Chromosome

c)

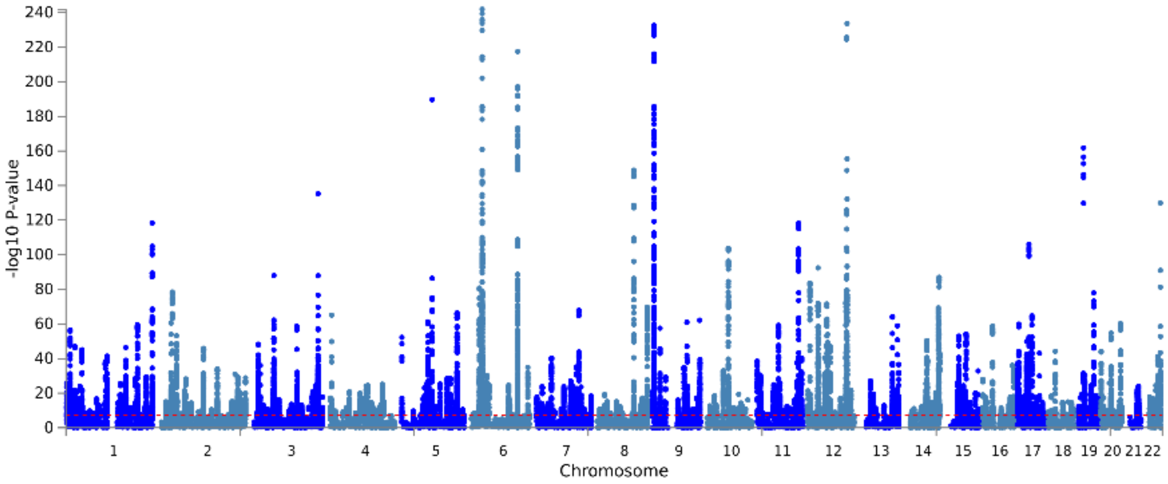

Figure S1. Manhattan Plots of multi-trait associations with a) MPV, b) PDW and c) Plt. The x-axis shows chromosomal position, and the y-axis shows association p-values on a –log10 scale. Red dashed lines represent the statistical significance thresholds, ($\alpha = 5 \times 10^{-8}$).

Legend: MPV = mean platelet volume; PDW = platelet distribution width; Plt = platelet count.

a)

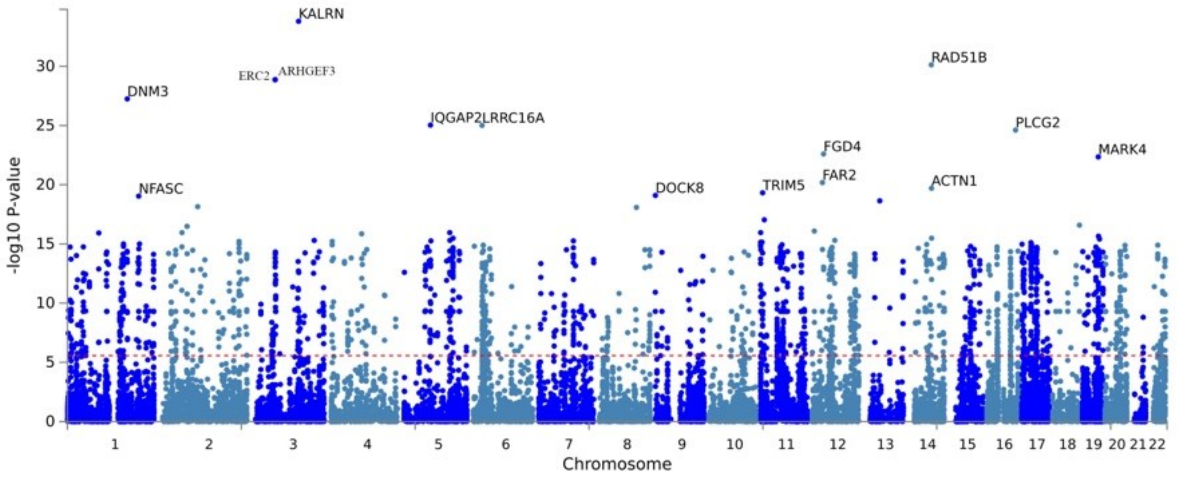
KALRN
RAD51B
30
ERC2
ARHGEF3
DNM3
25
IQGAP2
LRRC16A
PLCG2
FGD4
MARK4
FAR2
20
NFASC
DOCK8
TRIM5
ACTN1
-log10 P-value
15
10
5
0
1 2 3 4 5 6 7 8 9 10 11 12 13 14 15 16 17 18 19 20 21 22
Chromosome

b)

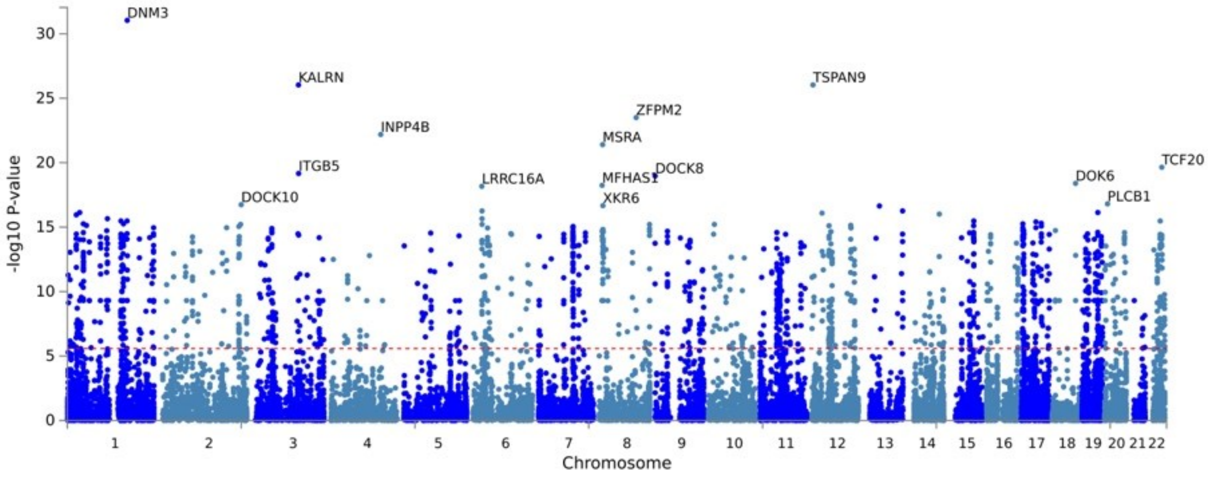
DNM3
30
KALRN
TSPAN9
25
ZFPM2
INPP4B
MSRA
20
ITGB5
DOCK8
TCF20
MFHAS1
LRRC16A
DOK6
DOCK10
XKR6
PLCB1
-log10 P-value
15
10
5
0
1 2 3 4 5 6 7 8 9 10 11 12 13 14 15 16 17 18 19 20 21 22
Chromosome

c)

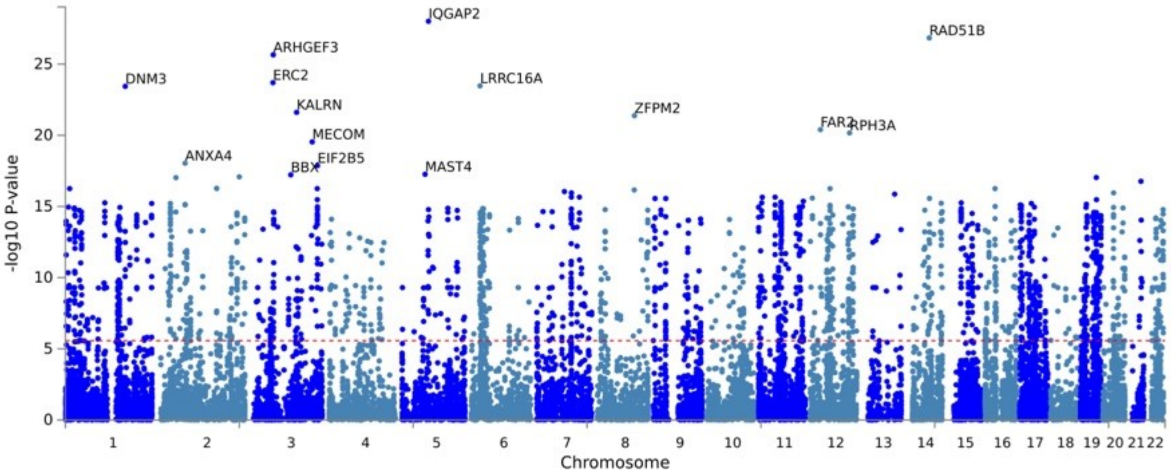

Figure S2. Manhattan Plots of gene-based enrichments of multi-trait associations with a) MPV, b) PDW and c) Plt. The x-axis shows chromosomal position, and the y-axis shows association p-values on a –log10 scale. Red dashed lines represent the statistical significance thresholds, (α =2.7×10^{-6}).

Legend: MPV = mean platelet volume; PDW = platelet distribution width; Plt = platelet count.

a)

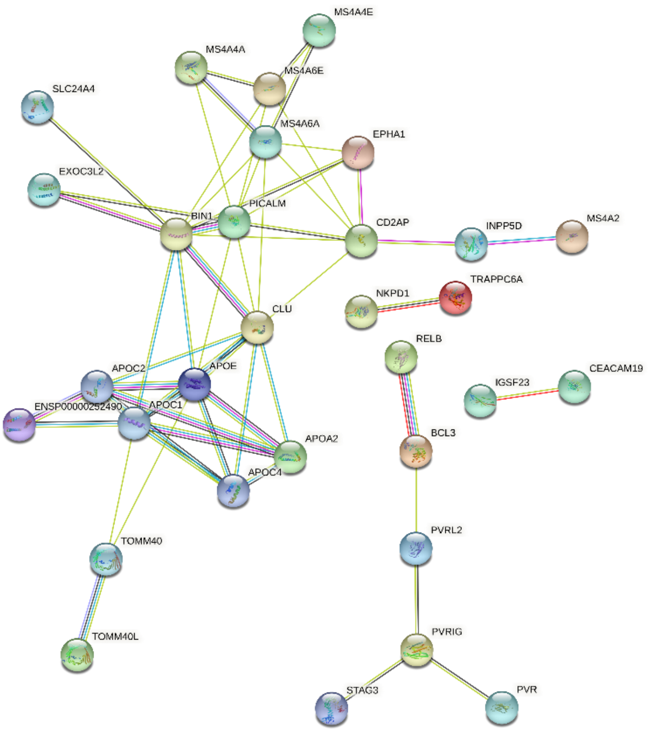
MS4A4E
MS4A4A
MS4A6E
SLC24A4
MS4A6A
EPHA1
EXOC3L2
PICALM
BIN1
CD2AP
INPP5D
MS4A2
TRAPPC6A
NKPD1
CLU
RELB
CEACAM19
IGSF23
APOC2
APOE
ENSP00000252490
APOC1
APOA2
BCL3
APOC4
PVRL2
TOMM40
TOMM40L
PVRIG
STAG3
PVR

b)

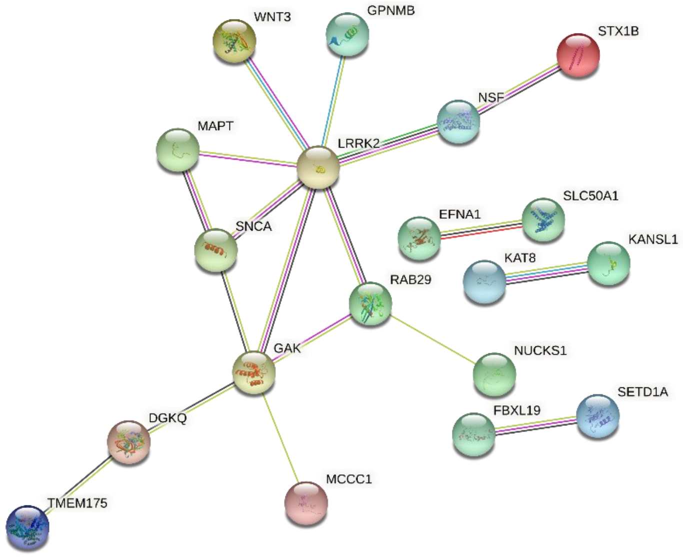
WNT3
GPNMB
STX1B
NSF
MAPT
LRRK2
SLC50A1
EFNA1
SNCA
KANSL1
KAT8
RAB29
GAK
NUCKS1
SETD1A
FBXL19
DGKQ
MCCC1
TMEM175

c)

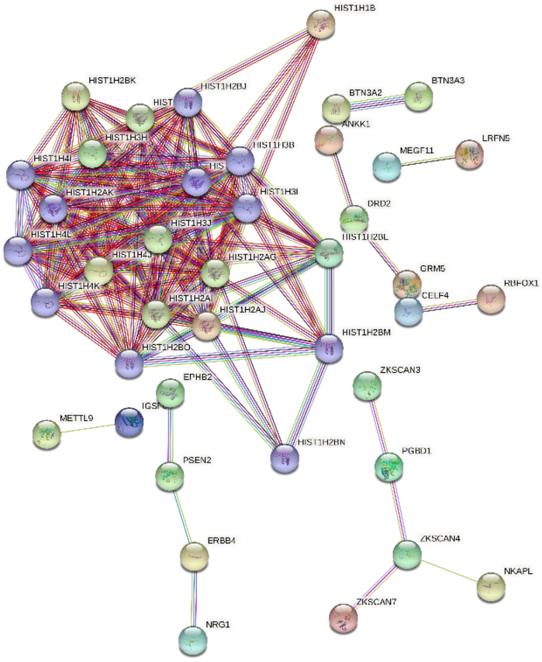

Figure S3. Protein-protein interaction network of genes significantly enriched for associations with a) AD, b) PD and c) MDD. The reported network - including both direct (physical) and indirect (functional) associations – was based on the STRING v11.0 database (Szklarczyk et al., 2019). Only high-confidence interactions between proteins are reported (interaction score > 0.7), while disconnected nodes in the network were hidden. Each node represents all the proteins produced by a single protein-coding gene locus, while edges represent protein-protein associations. Line color indicates the type of interaction evidence: light blue = from curated databases; purple = experimentally determined; green = gene neighborhood; red = gene fusions; blue = gene co-occurrence; yellow = text-mining; black = co-expression; violet = protein homology.

Legend: AD = Alzheimer's Disease; PD = Parkinson's Disease; MDD = Major Depressive Disorder.

Table S1. Network Statistics of protein-protein interaction networks of genes significantly enriched for associations with AD, PD and MDD.

	AD	PD	MDD	AD-PD-MDD
number of nodes:	57	63	120	240
number of edges:	57	18	264	281
average node degree:	2.00	0.57	4.4	2.34
avg. local clustering coefficient:	0.36	0.23	0.47	0.328
expected number of edges:	2	3	49	61
PPI enrichment p-value:	<1.0e-16	8.46E-09	<1.0e-16	< 1.0e-16

Network statistics for both separate lists of enriched genes for each disorder and for a single merged list of the three disorders – so to evaluate "global" interactions -are reported.

Legend: AD = Alzheimer's Disease; PD = Parkinson's Disease; MDD = Major Depressive Disorder.

Table S2. Significant gene-set enrichments of associations with a) AD, b) PD and c) MDD

a)

Gene Set_AD	N Genes	Beta(SE)	PBon
GO_bp:go_negative_regulation_of_amyloid_precursor_protein_catabolic_process	13	2.23(0.26)	2.74E-13
GO_bp:go_regulation_of_amyloid_beta_formation	21	1.35(0.21)	2.14E-06
Curated_gene_sets:roversi_glioma_copy_number_up	97	0.58(0.10)	4.01E-05
GO_cc:go_neurofibrillary_tangle	5	2.21(0.38)	6.43E-05
GO_bp:go_amyloid_beta_formation	27	1.06(0.19)	0.0001354
GO_bp:go_regulation_of_amyloid_precursor_protein_catabolic_process	27	1.02(0.18)	0.00016507
GO_bp:go_negative_regulation_of_metalloendopeptidase_activity	4	2.28(0.45)	0.00234972
GO_bp:go_amyloid_beta_metabolic_process	40	0.74(0.15)	0.00274215
GO_bp:go_amyloid_precursor_protein_catabolic_process	37	0.79(0.16)	0.00481566
GO_bp:go_regulation_of_aspartic_type_endopeptidase_activity_involved_in_amyloid_precursor_protein_catabolic_process	9	1.44(0.30)	0.00798928
GO_bp:go_negative_regulation_of_metallopeptidase_activity	5	2.11(0.43)	0.00806658
GO_bp:go_regulation_of_aspartic_type_peptidase_activity	11	1.27(0.27)	0.01465055
GO_mf:go_snare_binding	101	0.39(0.09)	0.04459249
Curated_gene_sets:busa_sam68_targets_dn	7	1.55(0.34)	0.04582395

b)

Gene Set_PD	N Genes	Beta(SE)	PBon
GO_mf:go_igg_binding	9	1.54(0.31)	0.00556697

c)

**Gene Set_MDD**	**N Genes**	**Beta(SE)**	**PBon**
GO_cc:go_gaba_ergic_synapse	64	0.74(0.13)	9.56E-05
GO_cc:go_intrinsic_component_of_synaptic_membrane	159	0.42(0.08)	0.00069426
GO_bp:go_branching_morphogenesis_of_a_nerve	10	1.64(0.31)	0.00164975
GO_cc:go_u6_snrnp	6	1.96(0.38)	0.00225066
GO_cc:go_lsm2_8_complex	6	1.96(0.38)	0.00225066
GO_cc:go_intrinsic_component_of_postsynaptic_density_membrane	52	0.68(0.14)	0.00434907
GO_cc:go_neuron_to_neuron_synapse	323	0.27(0.06)	0.01402979
GO_cc:go_intrinsic_component_of_postsynaptic_specialization_membrane	73	0.55(0.12)	0.01412962
GO_cc:go_postsynaptic_density_membrane	71	0.57(0.12)	0.0151148
GO_bp:go_synapse_assembly	160	0.37(0.08)	0.02770262
GO_cc:go_synaptic_membrane	400	0.23(0.05)	0.03671578

Legend: N Genes: number of genes detected for specific Gene Ontology; SE: standard error; PBon: p-value adjusted by Bonferroni correction.
